# Supplementary material for: Mechanisms governing the pioneering and redistribution capabilities of the non-classical pioneer PU.1
Source: Nat Commun. 2020 Jan 21;11:402. doi: 10.1038/s41467-019-13960-2 (PMC6972792; doi:10.1038/s41467-019-13960-2)
Supplement: Supplementary file 7 — Source data [file 41467_2019_13960_MOESM7_ESM.zip › Source_Data/FigureS3/FigureS3H_MotifScanOutput/homerResults/motif4.similar.html]

motif4

## Information for motif4

G
T
C
A
A
C
T
G
A
C
T
G
C
G
T
A
A
C
G
T
A
C
T
G
A
G
C
T
A
C
T
G
A
T
C
G
A
C
T
G
  
Reverse Opposite:  

T
G
A
C
A
T
G
C
A
G
T
C
C
T
G
A
A
G
T
C
C
G
T
A
A
C
G
T
A
G
T
C
A
G
T
C
A
C
G
T
  

|  |  |
| --- | --- |
| p-value: | 1e-136 |
| log p-value: | -3.150e+02 |
| Information Content per bp: | 1.924 |
| Number of Target Sequences with motif | 277.0 |
| Percentage of Target Sequences with motif | 7.17% |
| Number of Background Sequences with motif | 342.7 |
| Percentage of Background Sequences with motif | 0.76% |
| Average Position of motif in Targets | 262.2 +/- 193.1bp |
| Average Position of motif in Background | 197.5 +/- 127.3bp |
| Strand Bias (log2 ratio + to - strand density) | -0.1 |
| Multiplicity (# of sites on avg that occur together) | 1.00 |
| Motif File: | file (matrix) reverse opposite |

### Similar de novo motifs found

|  |  |  |  |  |  |  |  |
| --- | --- | --- | --- | --- | --- | --- | --- |
| Rank | Match Score | Redundant Motif | P-value | log P-value | % of Targets | % of Background | Motif file |
| 1 | 0.856 | C G T A C T A G T A C G T G C A C A G T A C T G C G A T C A T G C A T G A T C G C A G T A C G T A C G T A G C T | 1e-49 | -113.665580 | 11.30% | 4.96% | motif file (matrix) |
